# B7H4 Expression Is More Frequent in MSS Status Colorectal Cancer and Is Negatively Associated with Tumour Infiltrating Lymphocytes

**DOI:** 10.3390/cells12060861

**Published:** 2023-03-10

**Authors:** Miriam Dawidowicz, Agnieszka Kula, Sylwia Mielcarska, Paweł Kiczmer, Hanna Skiba, Małgorzata Krygier, Magdalena Chrabańska, Jerzy Piecuch, Monika Szrot, Julia Robotycka, Błażej Ochman, Bogumiła Strzałkowska, Zenon Czuba, Elżbieta Świętochowska, Dariusz Waniczek

**Affiliations:** 1 Department of Oncological Surgery, Faculty of Medical Sciences in Zabrze, Medical University of Silesia, 41-808 Ksatowice, Poland; 2 Department of Medical and Molecular Biology, Faculty of Medical Sciences in Zabrze, Medical University of Silesia, 19 Jordana, 41-800 Zabrze, Poland; 3 Department and Chair of Pathomorphology, Faculty of Medical Sciences in Zabrze, Medical University of Silesia, 13-15 3 Maja, 41-800 Zabrze, Poland; 4 Department of General and Bariatric Surgery and Emergency Medicine, Faculty of Medical Sciences in Zabrze, Medical University of Silesia, 41-808 Katowice, Poland; 5 Department of Microbiology and Immunology, Faculty of Medical Sciences in Zabrze, Medical University of Silesia, 40-055 Katowice, Poland

**Keywords:** B7H4, microsatellite stable tumours, iTME, CRC, immune checkpoint

## Abstract

The immunotherapies based on ICIs in CRC are nowadays limited to microsatellite unstable tumours which are approximately 15% of all CRC cases. There are a few new immune checkpoints belonging to the B7 family, including B7H4. B7H4 expression is associated with so-called “cold tumours”, and its function is linked to the downregulation of various immune cell populations. Our study aimed to investigate whether B7H4 expression is dependent on microsatellite status in CRC and on elucidating the immunological context in which the expression of B7H4 occurs. We enrolled 167 patients in the study. We prepared the homogenates from tumour tissues and healthy adjacent tissue to assess the B7H4 levels and the Bio-Plex Pro Human 48-cytokine panel. We assessed the microsatellite status of the tumour, B7H4 expression, CD8+ T cell population, and the TILs and budding in H + E stained slides by the IHC method. We used an online available database for further exploring the biological characteristics of B7H4. The expression of B7H4 was more frequent in microsatellite stable tumours, and was negatively associated with TILs. B7H4 is positively correlated with antitumour immunosuppressive iTME, thus contributing to the immunosuppressive environment in CRC.

## 1. Introduction

The B7H4 (aliases VTCN1, B7S1, B7x) protein belongs to the B7 family [1]. The B7 family ligands (present on APCs-Antigen presenting cells) bind to its counter receptor from the CD28 family (present on the T cell), which plays a central role in fine-tuning the antigen-specific immune response. This immune response belongs to cell-mediated adaptive immunity, which is particularly important in the proper antitumour response. B7H4 is a co-inhibitory ligand of the B7 family [2]. The general function of B7H4 is to downregulate immune reactions by inhibiting T-cell activation, proliferation, and cytokine production. The expression of B7H4 is limited in normal tissues; however, its overexpression has been confirmed in a wide selection of solid malignancies, including lung, liver, kidney, ovary, stomach, skin, pancreas, colorectal, and breast cancer [3,4,5,6,7]. 

The members of the B7 family include, among others, PD-L1, PD-L2, PD-1, and CTLA-4. These molecules have a highly important place in oncological clinical practice as targets for immunotherapy. An immune checkpoint blockade against them is used in a broad range of human malignancies [8]. However, they are not universal. In colorectal cancer, their usefulness is limited to patients with damage mismatch repair genes (dMMR), for whom microsatellite instability (MSI) is its marker. The group of patients with MSI status represents only approximately 15% of all patients suffering from colorectal cancer [9,10,11,12,13].

The selective upregulation of PD-1, CTLA-4, LAG-3, Tim-3, and killer immunoglobulin-like receptors is often present in MSI-H tumours. The presence of these co-inhibitory receptors may explain the phenomenon.

MSI-H tumours are not eliminated naturally, despite high immune activation in this type of cancer. Furthermore, tumour-infiltrating cells (TILs) express high levels of PD-1 in MSI colorectal cancer, which is absent in microsatellite stable tumours (MSS); this explains why a checkpoint blockade is effective in MSI status tumours [14,15]. For dMMR/MSI-H colorectal cancer, there are approved anti-programmed cell death protein 1 (PD-1) antibodies called pembrolizumab and nivolumab for treating patients who have previously received chemotherapy. Additionally, the combination of nivolumab with ipilimumab (a CTLA-4 inhibitor) to treat MSI-H colorectal cancers that progressed prior to chemotherapy is available [16].

The tumour microenvironment (TME) consists of stromal, extracellular components, and immune cells. The immune part creates only immunological tumour microenvironment (iTME). The iTME can vary across the cancer subtypes and the disease stage [17]. The iTME is a dynamic system in which the combination of cell types, location, and functional orientation leads to the creation of an effective anti-tumour barrier and further tumour rejection or a tumour-promoting environment. Cytokines are some of the most important components within the iTME and are involved in a conflict between tumour cells and tumour-infiltrating immune cells [18,19]. However, there are limited data on how the local cytokinome landscape influences the expression of B7H4 in colorectal cancer [20]. 

This study aimed to evaluate the expression of the B7H4 to MSI/MSS status and other clinicopathological features of CRC. Furthermore, another goal was to explain the immunological context in which the expression of B7H4 occurs, through the 48-cytokine screening panel of cancer tissues homogenates and immunogenic features, immune composition, and functional annotations analysis of online available datasets. 

## 2. Materials and Methods

### 2.1. Characteristics of the Patient Group

The samples from 167 patients obtained during surgery due to CRC were used in the study. Patients were treated in the 1st Specialist Hospital in Bytom, Poland (approval of the Research Ethics Committee No. KNW/0022/KB1/42/III/14/16/18, 14 July 2020). The collected specimens included colorectal tumour tissues and adjacent normal tissue. Patients were enrolled based on the inclusion and exclusion criteria described in our previous paper [21]. Research group characteristics are presented in Table 1.

### 2.2. Evaluation of the B7H4 Expression by ELISA

Fragments of the tumour tissue and surgical tissue margin were weighted and homogenized according to the standard homogenization protocol already described in our previous paper [21]. To assess the levels of the B7H4 protein, an enzyme-linked immunosorbent assay (ELISA) was used, following the manufacturer’s instructions. B7H4 levels were evaluated by a human B7H4 ELISA kit (Cloud Clone, Wuhan, China) with a sensitivity of 56 pg/mL. The absorbance of the samples was determined using a Universal Microplate Spectrophotometer (μQUANT, Biotek Inc., Winooski, VT, USA). The measurement was conducted at a wavelength of 450 nm. The obtained results were recalculated to the corresponding total protein level and presented as pg/mg of protein.

### 2.3. Evaluation of the B7H4 Expression by IHC

For B7H4 expression, the B7H4 immunostaining was performed in 76 cases in which the MSI/MSS statuses were also established. Tissue samples were obtained from formalin-fixed paraffin-embedded tissue blocks with primary CRC and tumour-free margin samples. Then, the samples were deparaffinized and rehydrated. In the next step, antigen retrieval was performed by incubating slices in EnVision Flex Target Retrieval Solution High pH (Dako, Carpinteria, CA, USA) for 20 min at 95 °C. Prepared samples were incubated with Peroxidase-Blocked Reagent (Dako) and then incubated with antibody: B7-H4 Polyclonal Antibody, Invitrogen, incubation time: 40 min.; dilution: 1:1500. After this process, they were put in EnVision FLEX HRP (Dako). Then, antigen–antibody complexes were stained using 3,3′-diaminobenzidine. Finally, tissue sections were counterstained with hematoxylin, dehydrated, and covered with coverslips for further analysis. 

### 2.4. Assessment of the MSI/MSS Status

For MSI/MSS status evaluation in 101 cases, the IHC staining for MSH2, MSH6, PMS2, and MLH1 was performed on 4 µm thick sections of a representative formalin-fixed, paraffin-embedded (FFPE) tumour tissue block on a Dako Autostainer Link 48. Samples underwent deparaffinization and rehydratation. In the next step, antigen retrieval was performed by incubating slices in EnVision Flex Target Retrieval Solution High pH (Dako, Carpinteria, CA, USA) for 20 min at 95 °C. Prepared samples were incubated with Peroxidase-Blocked Reagent (Dako) and then incubated with one of the following antibodies: Mouse Monoclonal antibody MSH2 (G219-1129), Cell Marque, incubation time: 30 min.; dilution: 1:400; Mouse Monoclonal antibody MSH6 (44), Cell Marque, incubation time: 45 min.; dilution: 1:100; Mouse Monoclonal antibody PMS2 (MRQ-28), Cell Marque, incubation time: 40 min.; dilution: 1:50; Mouse Monoclonal antibody MLH1 (G168-728), Cell Marque, incubation time: 40 min.; dilution: 1:100. After this process, they were put in EnVision FLEX HRP (Dako). Then, antigen–antibody complexes were stained using 3,3′-diaminobenzidine. Finally, tissue sections were counterstained with hematoxylin, dehydrated, and covered with coverslips for further analysis.

Tumours were assessed as to whether nuclear staining of invasive tumour cells for MSH2, MSH6, PMS2, and MLH1 was seen in the presence of positive internal control (inflammatory and stromal cells). Tumours with nuclear staining for markers in at least 1% of invasive tumour cells were considered to have positive marker staining. The algorithm on which the interpretation of immunohistochemistry testing was based is presented in the article of Olave and Graham [22]. The MSI status was recognized if one of the following marker layouts were present: MLH1 and PMS2 loss, PMS2 loss, MSH2, and MSH6 loss, or MSH6 loss. We assumed negative staining as the loss. 

### 2.5. Assessment of the Tumour-Infiltrating CD8+ T Cells 

Briefly, 4 µm thick tissue sections were used for immunohistochemical (IHC) analysis. They were deparaffinized with xylene, rehydrated in graded alcohol, and washed in deionized water. In the next step, antigen retrieval was performed by incubating slices in EnVision Flex Target Retrieval Solution High pH (Dako, Carpinteria, CA, USA) for 20 min at 95 °C. Prepared samples were incubated with Peroxidase-Blocked Reagent (Dako) and then incubated with antibody: (CD8+/144B) Mouse Monoclonal Antibody diluent, incubation time: 40 min.; dilution: 1:100. After this process, they were put in EnVision FLEX HRP (Dako). Then, antigen–antibody complexes were stained using 3,3′-diaminobenzidine. Finally, tissue sections were counterstained with hematoxylin, dehydrated, and covered with coverslips for further analysis.

### 2.6. Assessment of the TILs and Budding

Tumour-infiltrating lymphocytes (TILs) assessment was performed in 102 specimens. The percentage of tumour-associated lymphatic infiltration was estimated semi-quantitatively on a five-grade scale on the same H&E-stained slides by the two pathologists, according to the criteria defined by Salgado et al. in breast cancer [23]. These include intratumoural lymphocytes with cell-to-cell contact between lymphocytes and tumour cells, and stromal TILs in tumour tissue located dispersed in the stroma within the tumour cells without direct contact, including TILs at the invasive margin. According to the recommendations, stromal TILs were scored as a percentage of the stromal area alone, excluding areas occupied by carcinoma cells. Lymphatic infiltrates outside the tumour borders were not included in the evaluation. A lymphocyte infiltration area lower than 5% was considered TILs 1, whereas 5–25%, 25–50%, and 50–75% of lymphocytes in the stroma were defined as TILs 2, TILs 3 and TILs 4, respectively. More than 75% was defined as TILs 5.

Tumour budding was assessed in the same 101 specimens. Tumour buds were estimated in one FOV at a hotspot area in the invasive front under ×20 magnification. The number of buds was adjusted by the normalization factor (1.210). Budding was reported in the following manner: low budding: 0–4 buds; intermediate budding: 5–9 buds; high budding: >10 buds. The mean number of buds per FOV was also used in the statistical analysis.

### 2.7. Assessment of the Cytokines Screening Panel

Homogenates’ supernatants were collected after they were stored at −80 °C. The concentrations of cytokines/chemokines/growth factors were measured in 77 homogenates by the Bio-Plex Pro Human cytokines screening panel 48 cytokines assay (Bio-Rad Laboratories, Hercules, CA, USA) according to the manufacturer’s instructions. In brief, 50 µL aliquot of the sample was diluted 1:2 with sample diluent, incubated with antibody-coupled beads and biotinylated secondary antibodies, and followed by streptavidin-phycoerythrin. Standard curves for each studied parameter were performed using respective cytokine standard solutions. The beads were analysed in the Bio-Plex Array Reader (Bio-Plex Manager 6.2 software from the Bio-Plex 200 System). The intra-assay %CV varied up to 15%, and the inter-assay %CV varied up to 25%, depending on the analysed parameter. The obtained results were then normalised to the corresponding total protein level. This method has been previously used in analysing cytokines in lymphocyte cell culture supernatants and in blood serum samples [24,25]. 

### 2.8. Exploration of Biological Characteristics of B7H4

We performed functional annotations analysis based on mRNA expression profiles in the CRC online dataset from the FieldEffectCrc Package among cohort A, consistent with 311 CRC samples [26]. We normalized the matrix data using the DESeq2 package [27]. Then, we divided the cohort into high versus low expressions of VTCN1(B7H4). Gene set enrichment analysis was used to elucidate the potential Hallmarks pathways from The Molecular Signatures Database (h.all.v7.5.symbols.gmt) of B7H4 in CRC in R Studio with fgsea package. The genes with significant differences in expression, including high vs. low B7H4 expression, were screened for GO enrichment analyses (|logFC| > 0.5 and p.adj. < 0.05). Then, we used TCGA-COAD data to explore the association of B7H4 expression with immunogenicity and immune landscape in colorectal cancer. Results of the immune-related scores, mutation analysis, and immune cell infiltration scores, were all obtained from the CAMOIP website (http://220.189.241.246:13838/#shiny-tab-188 home (accessed on 15 December 2022)).

### 2.9. Statistical Analysis

Data distribution was determined using the Shapiro–Wilk test. The log transformation of the concentrations of the studied proteins provided a better fit for the Gaussian distribution. The data are presented as mean ± SD for the variables with normal distribution, and as median with interquartile range for the variables with non-normal distribution. To compare the tumour and margin concentrations, the paired Student’s *t*-test (for variables with a normal distribution) and Mann–Whitney U test (for variables with non-normal distribution) were used. Independent variables were also compared using the Student’s *t*-test and Mann–Whitney U test. Pearson’s coefficient or Spearman coefficient were used for assessing the relationships between the examined variables (for variables with normal and non-normal distribution, respectively). Tau-Kendalls’ Tau Rank Correlation Coefficient was used to determine the association between the levels of the examined proteins, T, and N parameters. *p* values < 0.05 were considered significant. Hierarchical clustering and principal component analysis were performed to reduce the number of variables and clarify the influence of B7H4 expression on the cytokines profile. The factors obtained in PCA were used as new variables named Dim 1 and Dim 2 according to their proportions of the explained variance. Next, the Dim 1 and Dim 2 values were used in further analyses. *p* values ≤ 0.05 were considered significant. Statistical analysis was performed using STATISTICA 13 software (Statsoft) and the R Studio (Integrated Development for R. RStudio, PBC, Boston, MA, USA).

## 3. Results

### 3.1. The Expression of B7H4 Is Upregulated in Tumour Tissues

**Figure 1:**
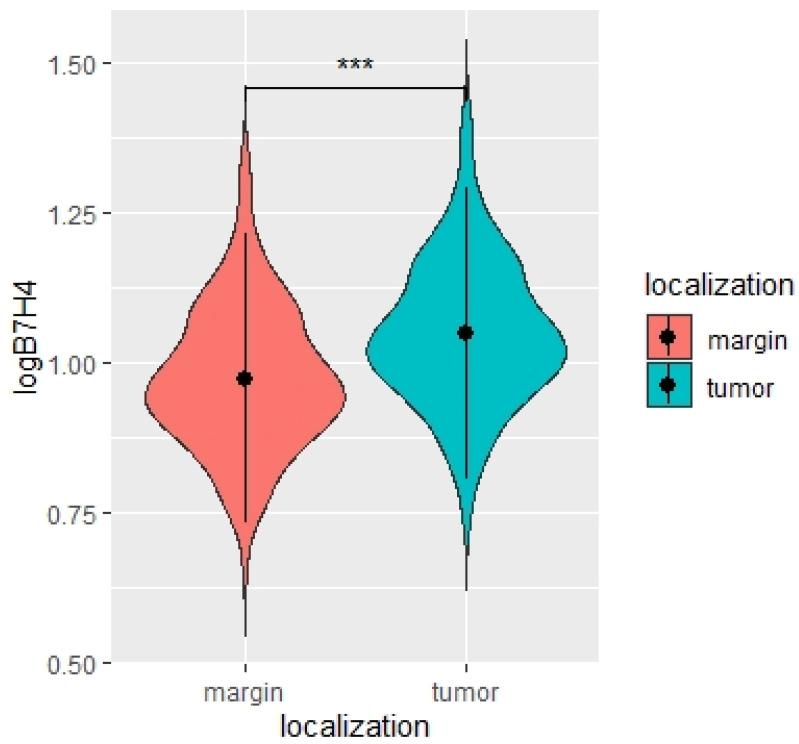
Violin plot of B7H4 concentrations in tumour and margin tissue [pg/mg]. Paired *t*-test. N = 159, *** *p* < 0.0001.

In this study, to elucidate the link between B7H4 expression, tumour microsatellite status, and the immunological background, the B7H4 expression level was first compared between tumour tissues and normal tissues adjacent to CRC. A total of 159 pairs of cancer tissues and normal adjacent tissues’ homogenates were analysed for B7H4 level, measured by ELISA. B7H4 was elevated in tumour tissues’ homogenates compared to normal adjacent tissues (*p* < 0.0001, Figure 1). 

**Figure 2:**
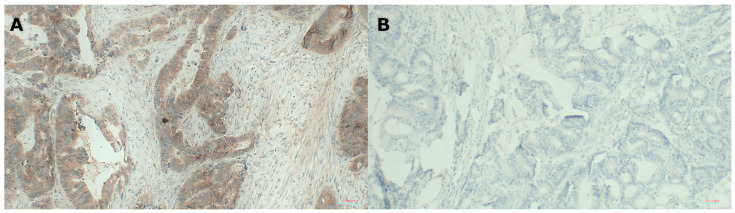
(**A**) Immunostaining for B7H4 positive tumour cells. (**B**) Immunostaining for B7H4 negative tumour cells. (Opta Tech 2200 Camera, magnification 200× and 100×).

We also analysed the expression of B7H4 by IHC in 76 tumour section slides. The percentage of B7H4 positive tumours was 78% (Table 2, Figure 2A).

### 3.2. The Expression of B7H4 Is Associated with the MSS Status of the Tumour

**Figure 3:**
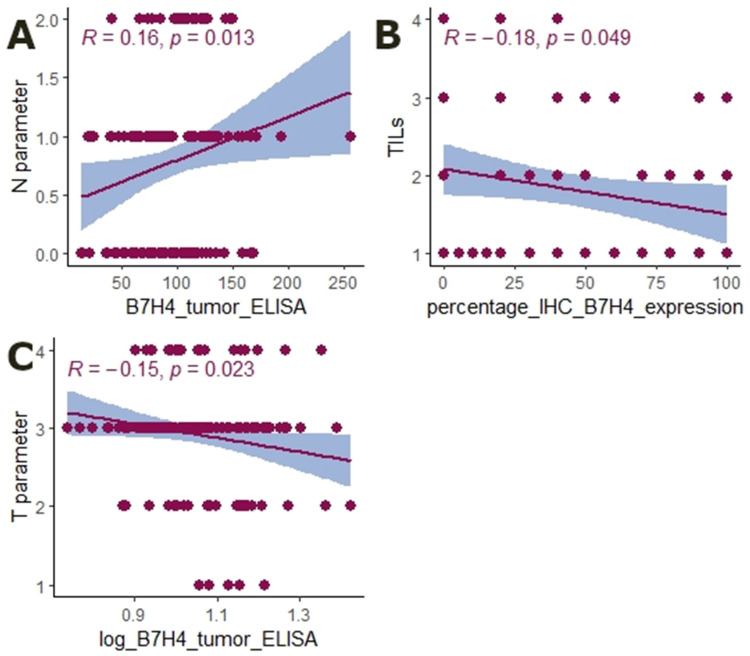
Graphical representation of Tau-Kendall correlation between B7H4 expression and clinicopathological parameters. (**A**) B7H4 [ng/mg], and N parameter (*p =* 0.013, R = 0.16, N = 152). (**B**) B7H4 IHC expression and TILs (*p =* 0.049, R = −0.18, N = 152). (**C**) B7H4 [ng/mg] and T parameter (*p* = 0.023, R = −0.15, N = 152).

Further, we analysed whether the expression of B7H4 is related to tumour microsatellite status. To elucidate this link we assessed the MSS/MSI status in 77 cases. B7H4 expression in the tumour was more frequent in MSS cases (*p =* 0.005, Table 2). Moreover, the tumour-infiltrating lymphocytes score was negatively correlated with the percentage of IHC B7H4 expression level (R = −0.18, *p =* 0.049, Figure 3). No association was found between CD8+ T cell infiltration and B7H4 expression. The B7H4 level was also weakly positively associated with the N parameter (R = 0.16, *p =* 0.013, Figure 3) and negatively with the T feature (R = −0.15, *p =* 0.023, Figure 3). There were no significant associations between B7H4 and other clinicopathological features (Table 2).

### 3.3. B7H4 Expression Is Negatively Associated with Pro-Inflammatory Cytokines

**Figure 4:**
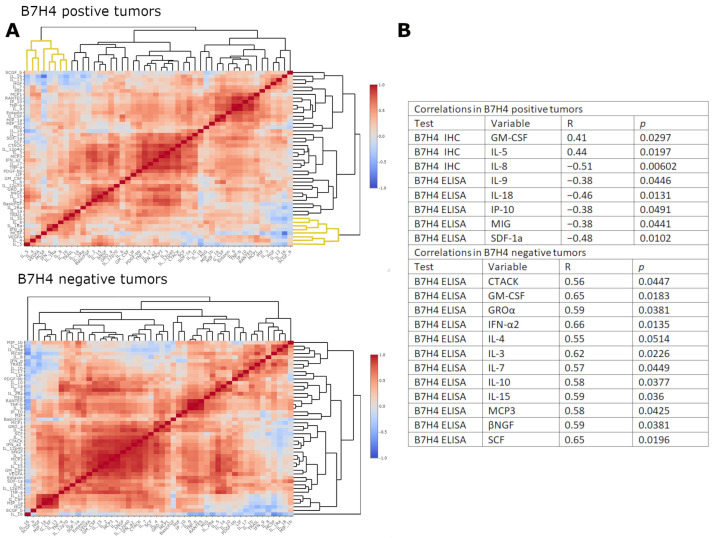
(**A**) Correlation heatmaps with dendrogram of 48 cytokines panel [pg/mg] in B7H4 tumour IHC positive cases and B7H4 tumour IHC negative cases. (**B**) Table of correlations between B7H4 measurements and cytokines in B7H4 IHC positive tumours, and between B7H4 ELISA measurements and cytokines in B7H4 IHC negative tumours (another source of B7H4 are immune cells) (Spearman correlation coefficient).

**Figure 5:**
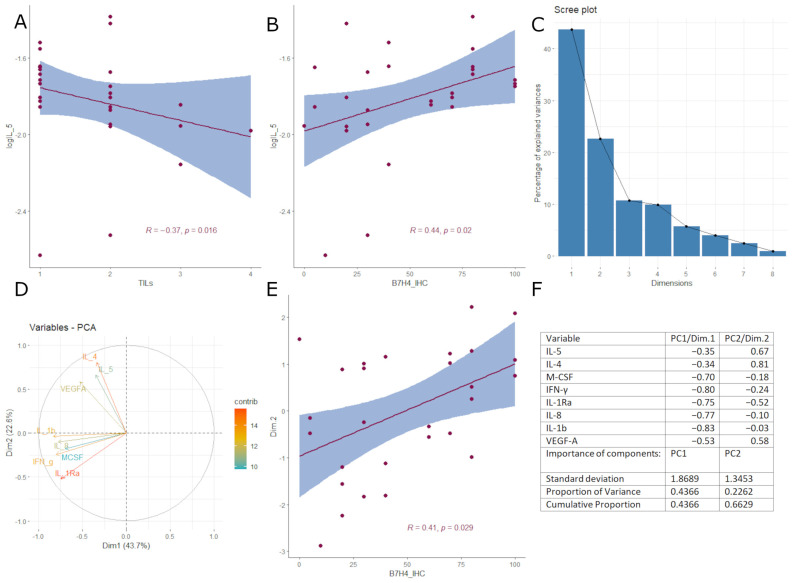
B7H4 positive tumours: (**A**) Correlation between IL-5 and B7H4 IHC expression (Spearman correlation coefficient) and (**B**) correlation between IL-5 and TILs (Tau-Kendall’s tau rank correlation coefficient); (**C**) Scree plot of the cluster chosen to PCA; (**D**) Principal Components representing variance in two dimensions; (**E**) Correlation between PC 2 (dim.2) and B7H4 IHC expression (Spearman correlation coefficient); (**F**) PCA component matrix for cytokines, Variables with strong correlative distribution (>0.5).

**Figure 6:**
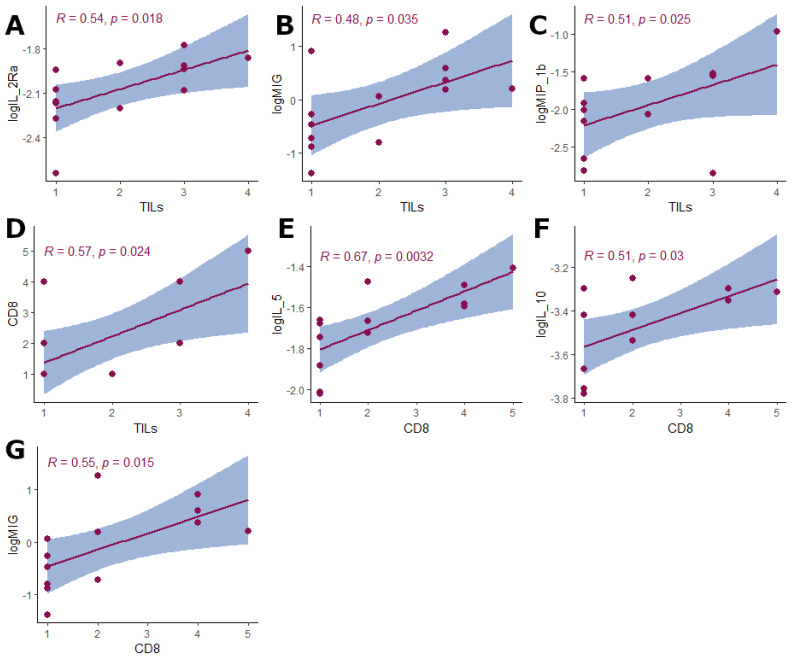
B7H4 negative tumours. (**A**)The correlation between Il-2Ra and TILs score *p* = 0.018, R = 0.54. (**B**) The correlation between MIG and TILs score *p* = 0.035, R = 0.48. (**C**) The correlation between MIP-1β and TILs score *p* = 0.025, R = 0.51. (**D**) The correlation between CD8+ T cells score and TILs score *p* = 0.024, R = 0.57. (**E**) The correlation between Il-5 and CD8+ T cells score *p* = 0.0032, R = 0.67. (**F**) The correlation between Il-10 and CD8+ T cells score *p* = 0.03, R = 0.51. (**G**) The correlation between MIG and CD8+ T cells score *p* = 0.015, R = 0.55 (Tau-Kendall’s tau rank correlation coefficient).

Next, we investigated the immunological background of B7H4 expression, the cytokinome composition in cancer tissue homogenates, to elucidate the cytokine differential expression pattern between B7H4 IHC positive and B7H4 negative tumours (Figure 4A). We divided our 48 cytokine panel by hierarchical clustering to create heatmaps of cytokine expression patterns in B7H4 positive and negative tumours. Heatmaps created two distinct expression patterns. The expressions of the following cytokines were inversely correlated with B7H4. ELISA measurements in B7H4 staining positive tumours: IL-9, IL-18, IP-10(CXCL10), MIG (CXCL9) and SDF-1a (CXCL12) (Figure 4B). Moreover, IL-5 alone correlated positively with B7H4 tumour staining percentage and inversely with TILs score (Figure 4 and Figure 5A,B). In B7H4 positive tumours, a cluster consisting of IL-5, IL-4, VEGFA, M-CSF, IFN-γ, IL-Ra, IL-8, and IL-1β was chosen for further analysis [20] (Figure 4A). We conducted Principal Component Analysis (PCA) with this cluster, obtaining two principal components that explained 66.28% of the overall variance (the sum of PC1 variance was 43.66% and PC2 variance was 22.62%) (Figure 5C,D,F). We found a positive correlation between PC2/Dim.2 and B7H4 IHC expression (Figure 5E). In the B7H4 positive group, no correlation between panel cytokines and CD8+ T cells infiltration was found; however, in the B7H4 negative group we found that IL-5, IL-10, and MIG positively correlated with CD8+ T cells (respectively, *p* = 0.0032, *p* = 0.03, *p* = 0.015 Figure 6E–G). In the same group, TILs were positively correlated with IL-2Ra, MIG, and MIP-1b (respectively, *p* = 0.018, *p* = 0.035, *p* = 0.025 Figure 6A–C).

### 3.4. Exploration of Immunogenicity and Immune Infiltration Landscape of B7H4 Based on TCGA-COAD Data

**Figure 7:**
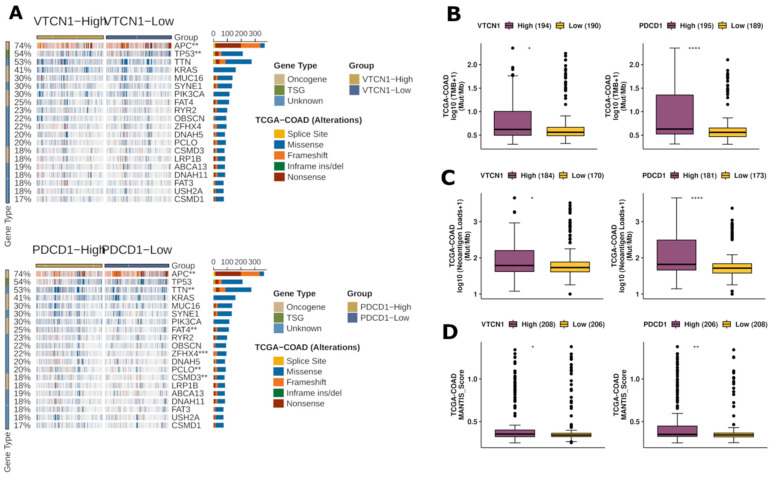
Immunogenicity-related analyses based on the TCGA-COAD dataset. (**A**) Differences in gene mutation frequencies between high and low PDCD1 expression groups and VTCN1(B7H4) high and low expression groups. (**B**) Tumour Mutational Burden comparison between VTCN1 expression groups and PDCD1. (**C**) Neoantigen Loads comparison between B7H4 expression groups and PDCD1. (**D**) Mantis score comparison between B7H4 expression groups and PDCD1. * *p* < 0.05, ** *p* < 0.01, and *** *p* < 0.001, **** *p* < 0.0001.

Subsequently, we further explored the effect of B7H4 on tumour immunity with online available tools. We juxtaposed the B7H4 immune landscape with the landscape of a known immune checkpoint in MSI type CRC PD-1 (*PDCD1*). Figure 7A shows the mutational landscape of gene mutations in B7H4 high vs. low expression and PD-1 high vs. low expression of CRC patients, indicating that PD-1 high has a higher frequency of mutations than B7H4 high. Except for the APC and TP53 genes, the other top 20 genes had higher mutation frequencies in the PD-1 high group. The types of mutations were mainly missense and frameshift mutations. The mutation frequencies of the APC and TP53 genes were higher in the B7H4 high group; in contrast, the other genes had higher mutation frequencies in the PD-1 high. In addition, Tumour Mutational Burden (TMB), and Neoantigen Loads (NAL) were significantly higher both in PD-1 high and B7H4 high groups (Figure 7B,C). However, the elevation of TMB and NAL scores in the B7H4 high was not as substantial as in PD-1 high group. Finally, the MANTIS score, which is a score that predicts a patient’s MSI status, was significantly higher in PD-1 high group. The higher the Mantis score, the more likely a patient will have an MSI-H status. These findings indicate that B7H4 expression is related to a moderately immunogenic landscape in CRC (Figure 7D). 

**Figure 8:**
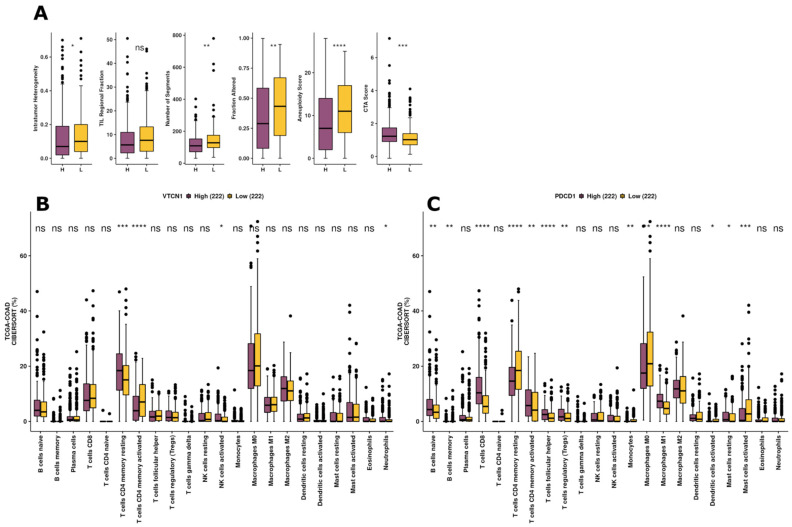
Differences in immune-related scores between different *VTCN1* and *PDCD1* expression groups. (**A**) Immune cell infiltration scores were calculated by the CIBERSORT algorithm in high and low *PDCD1* expression groups and *VTCN1* high and low expression groups. (**B**,**C**) Analyses based on the TCGA-COAD dataset, * *p* < 0.05, ** *p* < 0.01, and *** *p* < 0.001, **** *p* < 0.0001.

The immune scores of Intratumour Heterogeneity, TIL Regional Fraction, Number of Segments, Fraction Altered, and Aneuploidy Score were significantly up-regulated in the B7H4 low expression group (Figure 8A), unlike CTA Score, which was decreased in this group. When we compared immune cell infiltration scores calculated by the CIBERSORT algorithm in PD-1 high and B7H4 groups, we noticed that immune composition is in a greater portion shaped by PD-1 immune checkpoint expression. The diversity of iTME was slightly dependent on B7H4 expression. However, the fraction of CD4+ T cells was associated with B7H4 expression. In B7H4 high group, there was a significantly higher percentage of CD4+ memory resting T cells population and, on the other hand, the lower B7H4 expression was associated with a higher percentage of CD4+ memory activated T cells (Figure 8B,C). 

### 3.5. Functional Annotations and Predicted Signalling Pathways for VTCN1 (B7H4) Expression

**Figure 9:**
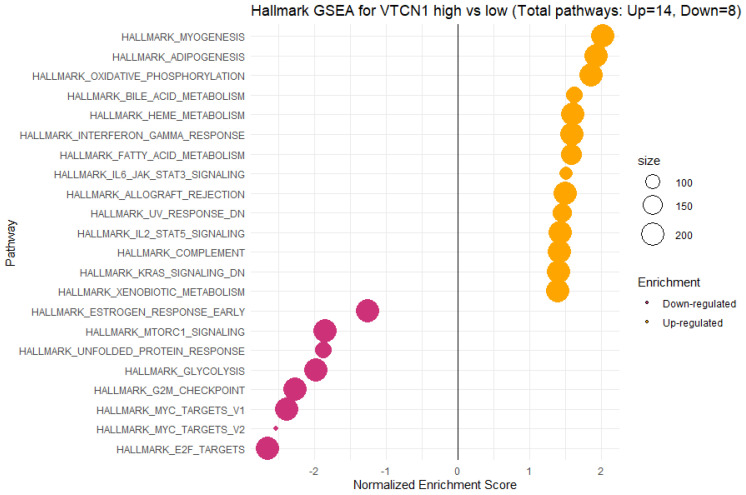
The Gene Set Enrichment Analysis (GSEA) of the relationship between the expression level of VTCN1(B7H4) in the FieldEffectCrc dataset. The most involved significant hallmark pathways were closely correlated with VTCN1 (B7H4) in CRC obtained by GSEA. NES: normalized enrichment score.

For further investigation of the B7H4 relation with transcriptome activity in CRC, we conducted GSEA between low and high B7H4 expression datasets to identify the B7H4-related pathways activated in CRC. Significant differences (*p.adjusted <* 0.05) in the enrichment of the Molecular Signature Database Collection for Hallmark gene sets are shown in Figure 9. The results showed that the top three upregulated pathways for B7H4 were myogenesis, adipogenesis, and oxidative phosphorylation. Furthermore, among upregulated pathways, there were genes upregulated by STAT5 in response to IL-2, genes encoding components of the complement system, which is part of the innate immune system, genes upregulated by IL-6 via STAT3, IFN-γ response, and genes downregulated by KRAS activation. These results showed that a high expression of B7H4 was closely associated with immune responses and malignancy in CRC. Conversely, a high expression of B7H4 has related downregulated pathways associated with the control of the cell cycle. Precisely, the first four most impacted and downregulated pathways were E2F Target, c-MYC, and G2/M Checkpoint, which suggested that the main effect is an impaired cell cycle block, particularly a G2/M phase transition arrest. 

**Figure 10:**
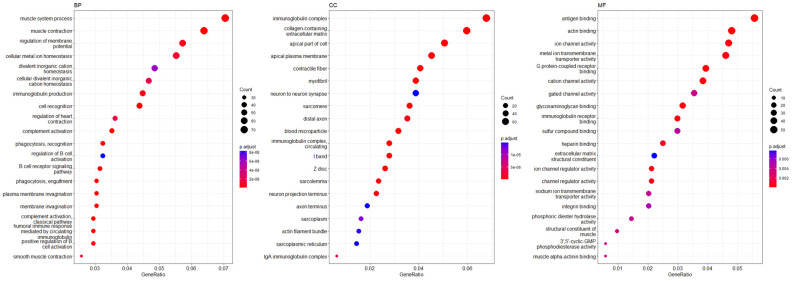
Significantly enriched GO annotations of VTCN1(B7H4) in the FieldEffectCrc dataset. BP: biological processes; CC: cellular components; MF: molecular functions.

Functional analyses of B7H4 demonstrated that the significant GO terms are mainly associated with the regulation of the muscle and related to immunological processes. We found that, in the biological process category, B7H4 expression was tightly associated with the regulation of B cell activation, immunoglobulin production, cell recognition, complement activation, and phagocytosis recognition, and was associated in the molecular function category with antigen binding (Figure 10).

## 4. Discussion

In this study, we provide the data of B7H4’s role in the immunological response in CRC. Firstly, we performed extensive research on the literature, and based on it we have chosen the B7H4 as the most probable immune checkpoint to be associated with the MSS type CRC. We assessed that B7H4 expression is higher in tumour tissue than in normal adjacent tissue, and is associated with MSS type CRC. Further, we investigated the relation of B7H4 positive and negative tumours to cytokines and we found out that the B7H4 is inversely correlated with antitumour cytokines. Subsequently, the analysis of the immune cell composition associated with a high expression of B7H4 showed the association with CD4+ T cells population. Finally, the GSEA and GO analyses were performed to elucidate what pathways the expression of B7H4 is related to. 

The B7H4 in recent years, as well as other immune checkpoints, has gained significant attention. There are a few reasons for this fact. Although the receptor for B7H4 remains unrecognized, B7H4, with the growing knowledge about its function, becomes an attractive therapeutic potential not only in the cancer field [28], but also in the autoimmune diseases field [2]. In normal tissues, human B7H4 mRNA is commonly expressed; however, B7H4 protein exhibits minimal or negative expression, e.g., in the intestine [29]. In contrast, its elevated expression has been observed in many tumour tissues [30]. In line with these findings, we also observed a higher expression of B7H4 in tumour tissue, and 78% positive stained tumours for B7H4 in our cohort. Similarly, Yan et al. reported 76.38% B7H4 positive tumour tissues in CRC [31]. The upregulation of B7H4 was also demonstrated in colorectal cancer in other studies [32,33]. Moreover, we found that the B7H4 positive tumours were significantly more frequent in microsatellite stable tumours. 

We further investigated the association between clinicopathological features and B7H4. The results showed a negative correlation between B7H4 expression and immune infiltrating cells (TILs), as we had expected. Tumour-Infiltrating Lymphocytes and CD8+ T lymphocyte density has been reported to be inversely correlated with B7H4 expression in many solid tumours [34,35,36]. However, we did not find an association between CD8+ T cell infiltration and B7H4 expression. On the other hand, Yan et al. found that B7H4 positively correlated with CD8+ T cells and negatively correlated with M2 macrophages and Treg cells. We found further contradictory results, as the B7H4 level was weakly negatively correlated with the T feature and positively associated with the N parameter. Another study reported that B7H4 was positively correlated with lymph node metastasis, advanced TNM stage, and poor tumour differentiation in CRC [31]. Although there are a few studies regarding clinicopathological features and their relation to B7H4, due to the inconclusiveness of their results we need to conduct further research on a larger cohort.

Understanding the tumour type-specific cytokine profile could provide insights into iTME targeted therapy using the plasticity of pro-tumour or anti-tumour polarised immune cells. The iTME targeted drugs could change the immunosuppressive environment of “cold tumours” to “hot” tumours and restore proper antitumour response [37]. To elucidate the cytokinome relation to the B7H4 tumour phenotype, using the hierarchical clustering method, we found out that the B7H4 tumour positive and B7H4 negative cytokinomes were significantly different. In a subsequent analysis of the B7H4 positive group, we observed a negative correlation between IL-9 and IL-18. IL-9, a Th2 cell cytokine, has been reported as one of the cytokines which are downregulated by B7H4 in lung cancer [38]. In CRC, its relation to B7H4 has not been yet investigated; however, studies show that the level of IL-9 decreased in CRC, along with the progression of the disease, and recent experiments confirmed the antitumour role of IL-9 [39,40,41,42]. Like IL-9, IL-18 also exhibits antitumour properties and its lower expression in CRC has been shown by Feng et al. They also observed that the upregulation of IL-18 leads to the inhibition of colon cancer cell proliferation [43]. CXCL10, CXCL9, and CXCL12 were also negatively correlated with B7H4. CXCL10 and CXCL9 are antitumour chemokines that can inhibit cancer cell proliferation as well as regulate immunity to recruit a variety of immune cells to kill tumour cells [44]. The role of CXCL12 in CRC is, on the other hand, contradictory and its interplay in iTME is more context-dependent; however, current knowledge is more prone to its pro-tumoral effect [45]. Another interesting finding was a positive correlation of IL-5 with B7H4 tumour staining percentage and its negative association with TILs score. The IL-5 main production sources are CD4+ T cells and type 2 innate lymphoid cells (ILC2s); the others are innate immune cells, such as mast cells and eosinophils [46]. In CRC, Th2 cells are considered to be the main source of IL-5 in iTME [20]. In breast cancer, an immune checkpoint blockade increased IL-5 production by CD4+ T cells [47]. We conducted PCA in the B7H4 positive group on the chosen cluster and we found a positive correlation between PC2 and B7H4 IHC expression. PC2 had the main contribution of IL-5, IL-4, and VEGF-A. Th2 immunosuppressive effect on iTME is exhibited by, among others, the secretion of IL-5 and IL-4 [20,48]. In the B7H4 positive group, we did not find any positive correlation between the cytokines panel, CD8+ T cells infiltration, and TILs. However, in the B7H4 negative group, we found a few cytokines that positively correlated with CD8+ T cells and TILs score. Thus, we may suspect that the main immunosuppressive effect of B7H4 on iTME comes from the tumour cells that express B7H4.

After exploring the effect of B7H4 on tumour immunity, we noticed that the mutational landscape of B7H4 high tumours was significantly different compared to the PD-1 related gene mutation load. Moreover, B7H4 expression exhibits a relation to the high mutation frequencies of the APC and TP53 genes, which were not observed in the PD-1 group. A highly similar mutational phenotype was observed by Lin et al. in the MSS/MSI-L CRC group [49]. Immunogenic factors such as Tumour Mutational Burden (TMB) and Neoantigen Loads (NAL) were slightly higher in the B7H4 high group, but not as high as in the PD-1 high group. These findings indicate that the expression of both immune checkpoints is related to a more immunogenic environment, despite the substantially more immunogenic tumour characterizing the PD-1 expression. Like in our results, the overall lower TMB and NAL scores were also reported in the MSS/MSI-L CRC group [49]. 

Intratumour Heterogeneity, Number of Segments, Fraction Altered, and Aneuploidy Score, were significantly lower in the B7H4 high expression group. Intratumour Heterogeneity (ITH) reversely association with B7H4 could be explained by results that link ITH levels with MSI status and, conversely, its lower score with CMS2 CRC subtype, which corresponds to the canonical epithelial tumour harbouring high chromosomal instability in a microsatellite stable (MSS) context [50,51]. Along with the Number of Segments, Fraction Altered, and Aneuploidy Score, the results for B7H4 expression are likely related to genomic stable tumours. According to Thorsson et al., the scores measuring DNA damage are related to immune infiltration across many cancer types and subtypes, including CRC [17]. Then, we investigated the immune composition of TME, beyond the TILs score and CD8+ T cell fraction measured in our cohort, on the TCGA-COAD dataset with the CIBERSORT algorithm. The results of that analysis revealed that the B7H4 expression influence on shaping the iTME is not very impactful, especially when compared to the role of PD-1 expression on immune composition. However, the B7H4 high group had a significantly higher percentage of T cells, CD4+ memory resting population and, on the contrary, a lower percentage of T cells, CD4+ memory activated. B7H4 suppressive effects on CD4+ and CD8+ T cells have been demonstrated in in vitro studies, where they exhibited the ability to suppress T cell effector functions, including inflammatory cytokine production and cytolytic activity [52,53]. Similar effects have been reported in a few cancer studies [54,55,56]. We have found that B7H4 is involved in the regulation of the CD4+ T cells effect in iTME, but we have not found its link to the CD8+ T cells population. It could be due to the overall lower tumour lymphocyte infiltration in B7H4 high expression tumours. However, along with the results from the cytokines panel, we suspect that the immunosuppressive role of B7H4 is the effect of the interplay with Th2 cells and the Th2 cytokines. Nevertheless, further investigation of iTME in B7H4 positive tumours is required to elucidate its exact effect on the immunological landscape in CRC. 

Finally, we used GSEA to survey the transcriptome of CRC cases to compare B7H4 high-expressed CRC versus low-expressed. The results showed that among the upregulated immune-related pathways, there were genes upregulated by STAT5 in response to IL-2, genes encoding components of the complement system, which are part of the innate immune system, and genes upregulated by IL-6 via STAT3, and IFN-γ response. IL-6/STAT3 as well as the IL-2/STAT5 pathway activates downstream target genes to protect tumour cells from apoptosis, increase tumour cell proliferation, cell cycle progression, invasion, and metastasis, and are involved in drug resistance [57,58]. Similar results for the B7H4 high expression group were observed in ovarian cancer [59]. Additionally, the downregulated pathways associated with the control of cell cycle at E2F Target and G2/M Checkpoint are related to the high expression of B7H4. In detail, E2F Target, c-MYC, and G2/M Checkpoint were the first four most impacted and downregulated pathways, suggesting that the main effect is an impaired cell cycle block, in particular a G2/M phase transition arrest. E2F is an essential protein that regulates the cell cycle with a block at the G2/M phase [60,61,62]. On the other hand, downregulated c-MYC target pathways could seem contradictory as c-MYC is an oncogene; however, this pathway includes many genes involved in restraining cell growth [62]. Functional analyses of B7H4 also confirmed its association with immunological-related processes. Significantly enriched GO terms referred to a wide range of immunological functions, from adaptive, with a predominance of the humoral response, to innate immunity, such as the regulation of B cell activation, immunoglobulin production, cell recognition, complement activation, phagocytosis recognition, phagocytosis engulfment, and antigen binding. These results indicate that B7H4 expression is linked to several pro-tumorigenic immunological processes and impaired cell cycle regulatory machinery. 

## 5. Conclusions

In conclusion, the findings of this study revealed that B7H4 expression is upregulated in CRC and is associated with the MSS status of tumours. Furthermore, B7H4 may play a pivotal role in the shaping of iTME in the microsatellite stable consistent immune landscape. B7H4 is positively correlated with antitumour immunosuppressive iTME. The iTME in the presence of B7H4 positive tumours might be mainly shaped by Th2 cells and their cytokine network. Additionally, the B7H4 positive tumour’s genomic landscape is similar to the one of MSS type CRC, which supports our finding that B7H4 expression is associated with microsatellite stable tumour types with a preserved mismatch repair gene mechanism. Taken together, the data suggest that B7H4 might be a potentially promising therapeutic target in MSS type CRC.

## Figures and Tables

**Table 1:** Patients’ characteristics (whole study group).

	Female	Male	All Cases
	76 (45.51%)	91 (54.49%)	167 (100%)
Age	66.63 ± 9.48	63.75 ± 9.46	65.09 ± 9.55
Tumour localization			
Left-sided	49 (66.22%)	65 (73.86%)	114 (70.37%)
Right-sided	25 (33.78%)	23 (26.13%)	48 (29.63%)
T parameter			
T1	1 (1.33%)	7 (8.05%)	8 (4.94%)
T2	16 (21.33%)	12 (13.79%)	28 (22.22%)
T3	47 (62.67%)	54 (62.07%)	101 (62.35%)
T4	11 (14.67%)	14 (16.09%)	25 (15.43%)
N parameter			
N0	32 (42.67%)	37 (42.05%)	69 (42.33%)
N1	30 (40.00%)	37 (42.05%)	67 (41.10%)
N2	13 (17.33%)	14 (15.91%)	27 (16.56%)
M parameter			
M0	66 (88.00%)	69 (79.31%)	135 (83.33%)
M1	9 (12.00%)	18 (20.69%)	27 (16.67%)
TNM stage			
I	13 (17.33%)	13 (14.77%)	26 (15.95%)
II	19 (25.33%)	21 (28.86%)	40 (24.54%)
III	34 (45.33%)	37 (42.05%)	71 (43.55%)
IV	9 (12.00%)	17 (19.32%)	26 (15.95%)
Grading			
Low	63 (85.14%)	75 (85.23%)	138 (85.16%)
High	11 (14.86%)	13 (14.777%)	24 (14.81%)
Adjuvant treatment			
Yes	7 (9.21%)	14 (15.38%)	21 (12.35%)
No	69 (90.79%)	77 (84.62%)	149 (87.65%)

**Table 2:** Association between clinicopathological parameters with B7-H4 IHC expression in tumours (N = 77).

Characteristics	B7H4 Tumour Expression		
	Positive	Negative	*p*
Age	63.82	67.88	0.13
Tumour localization			0.18
Left-sided	48 (81.36%)	11 (18.64%)	
Right-sided	12 (66.67%)	6 (33.33%)	
T parameter			0.67
T1	4 (100%)	0 (0%)	
T2	8 (80%)	2 (20%)	
T3	41 (78.85%)	11 (21.15%)	
T4	7 (70%)	3 (30%)	
N parameter			0.14
N0	25 (69.44%)	11 (30.56%)	
N1	25 (89.29%)	3 (10.71%)	
N2	10 (83.33%)	2 (16.67%)	
M parameter			0.91
M0	51 (78.46%)	14 (21.54%)	
M1	8 (80%)	2 (20%)	
TNM stage			0.20
I	10 (83.33%)	2 (16.67%)	
II	14 (63.64%)	8 (36.36%)	
III	28 (87.50%)	4 (12.50%)	
IV	8 (80%)	2 (20%)	
Grading			0.91
Low	51 (78.46%)	14 (21.54%)	
High	8 (80%)	2 (20%)	
MSS/MSI status			0.005
MSS tumours	56 (83.58%)	11 (16.42%)	
MSI tumours	4 (40%)	6 (60%)	
TILs			0.42
0–5%	30 (83.33%)	6 (16.67%)	
6–25%	18 (85.71%)	3 (14.29%)	
26–50%	10 (62.50%)	6 (37.50%)	
51–75%	2 (66.67%)	1 (33.33%)	
76–100%	0 (0%)	0 (0%)	
CD8 Lymphocytes			0.54
0–5%	27 (79.41%)	7 (20.59%)	
6–25%	17 (80.95%)	4 (19.05%)	
26–50%	10 (83.33%)	2 (16.67%)	
51–75%	3 (50%)	3 (50%)	
76–100%	3 (75%)	1 (25%)	
Budding			0.93
0–4	32 (80%)	8 (20%)	
5–9	18 (78.26%)	5 (21.74%)	
>9	9 (75%)	3 (25%)	

## Data Availability

Dampier CH (2020). “FieldEffectCrc: Tumor, tumor-adjacent normal, and healthy colorectal transcriptomes as SummarizedExperiment objects.” https://bioconductor.org/packages/release/data/experiment/html/FieldEffectCrc.html (accessed on 15 November 2022).
